# Ayurveda: The time to experiment

**DOI:** 10.4103/0974-7788.59935

**Published:** 2010

**Authors:** M. S. Valiathan, Urmila Thatte

**Affiliations:** *National Research Professor, Manipal University, Manipal, India*; 1 *Prof. and Head, Department of Chemical Pharmacology, Seth G S Medical College, Parel, Mumbai, India. E-mail: msaini@ualberta.ca*

The practice of medicine has a history of three thousand years in countries as varied as India, China, Egypt and Greece; but research in medicine, as understood today, dates back to no more than the time of Renaissance in Europe. The new features of research in Europe were accurate observation and experiment, which had their glorious exemplars in Vasalius and Harvey. Driven by faith in Atharvavedic period (1500 BC) and by empiricism during Buddhist centuries, Ayurveda became reason-based by the first century, when Charaka flourished. However, spared of Renaissance, the process of accurate observation and experiment did not enter India until the East-West encounter from sixteenth century, when pioneers such as Garcia da Orta and Van Rheed made detailed observations on India's medicinal plants. The taxonomic reports laid the foundation for pharmacological studies on medicinal plants, which were led by Sir RN Chopra in the twentieth century. Ever since, herbal drugs have occupied the center stage of Ayurvedic research in academia and industry in India.[1] By contrast, reports on clinical trials of drugs and procedures in compliance with the liberalized guidelines of WHO have been few; fewer still are studies based on the methods of modern science on Ayurvedic concepts and procedures. Admittedly, the application of current methods of Science to ancient concepts and procedures would bristle with difficulties.[2] To begin with, one would be faced with the problem of choosing subjects from among many in traditional medicine, which would lend themselves to interrogation by scientific methods. For example, the postulated homology between the components of the human body and those of the universe, equilibrium between the body and the environment, and scores of similar concepts are far from easy to rephrase as study questions in modern Science. Secondly, the use of controls and placebos, so central to clinical trials, would sit ill with Ayurveda, which regards every individual as unique, and the package of therapeutic measures including virtuous conduct, life style, diet, procedures and drugs as inseparable. The list could go on and reinforce the “Berlin wall” which has separated Ayurveda from modern Science for so long to mutual impoverishment.

The fact is that the use of modern Science as a research tool in Ayurveda has never been more necessary, more promising, or more compelling than at the present time. It is well known that India has over half a million “registered medical practitioners” who practice traditional medicine-not necessarily Ayurveda- and provide the back bone of health care in rural India. The Ayurvedic colleges graduate over 20,000 physicians every year to meet soaring demand, when Government has resolved to “mainstream Ayurveda” in healthcare. Traditional practices in Ayurveda such as *Panchakarma* have become so popular that Ayurvedic institutions regularly report waiting lists for patients from India and abroad. To ignore the testimony of thousands of patients over many decades is reminiscent of the derisive attitude of Jenner's contemporaries in Gloucestershire who despised the claim of milkmaids that cowpox gave them protection from small pox! When Jenner wrote to his mentor, John Hunter, on the observed fact and the arguments against it, Hunter gave his famous reply “Why think? Why not experiment?”. That applies to Ayurveda, whose time to experiment has arrived. Recent experience in India has shown that a science initiative can successfully formulate research projects on classic Ayurvedic topics such as *doshaprakriti, panchakarma* and *rasayana*, and involve scientists and Ayurvedic experts in well-planned, collaborative studies. Early observations have even suggested that the identification of phenotypes among populations based on ancient criteria could facilitate the correlation of personal phenotypes which personal genotypes. Basic studies of this kind have given a new dimension to research in Ayurveda without detriment to the ongoing work on herbal drugs.

Scientific studies cannot make progress without the publication of research findings, which must welcome and withstand the scrutiny of peers. Research in Ayurveda is, however, seriously handicapped by the paucity of peer-reviewed journals which would appeal as much to scientists as to the Ayurvedic community. We hope that this journal would play a significant role in filling the gap and advancing the cause of research in Ayurveda.
